# Discharging Women with Advanced Ovarian Cancer on Home Parenteral Nutrition: Making and Implementing the Decision

**DOI:** 10.3390/nu12010166

**Published:** 2020-01-07

**Authors:** Anne Marie Sowerbutts, Simon Lal, Jana Sremanakova, Andrew R. Clamp, Gordon C. Jayson, Antje Teubner, Lisa Hardy, Chris Todd, Anne-Marie Raftery, Eileen Sutton, Sorrel Burden

**Affiliations:** 1 Faculty of Medicine, Biology and Health, and Manchester Academic Health Science Centre, University of Manchester, Oxford Road, Manchester M13 9PL, UK; Jana.Sremanakova@manchester.ac.uk (J.S.); Andrew.Clamp@christie.nhs.uk (A.R.C.); Gordon.Jayson2@christie.nhs.uk (G.C.J.); Chris.Todd@manchester.ac.uk (C.T.); Sorrel.Burden@manchester.ac.uk (S.B.); 2 Salford Royal NHS Foundation Trust, Manchester M6 8HD, UK; Simon.Lal@srft.nhs.uk (S.L.); antje.teubner@srft.nhs.uk (A.T.); 3 The Christie NHS Foundation Trust, Manchester M20 4BX, UK; AnneMarie.Raftery@christie.nhs.uk; 4 Wythenshawe Hospital, University Hospital of South Manchester, Manchester University NHS Foundation Trust, M23 9LT Manchester, UK; lisa.hardy@mft.nhs.uk; 5 Population Health Sciences, Bristol Medical School, University of Bristol, Bristol BS8 2PS, UK; E.Sutton@bristol.ac.uk

**Keywords:** decision-making, parenteral nutrition, home, ovarian neoplasms, qualitative research

## Abstract

Increasingly, patients with advanced ovarian cancer in bowel obstruction are receiving home parenteral nutrition (HPN). Little is known about making and implementing the decision. This study explored the decision-making process for HPN and investigated the barriers and facilitators to implementation. This was a qualitative study underpinned by phenomenology involving 93 longitudinal in-depth interviews with 20 patients, their relatives and healthcare professionals, over 15 months. Participants were interviewed a maximum of four times. Interview transcripts were analysed thematically as per the techniques of Van Manen. We found variance between oncologists and patients regarding ownership of the HPN decision. The oncologists believed they were engaging in a shared decision-making process. However, patients felt that the decision was oncologist-driven. Nevertheless, they were content to have the treatment, when viewing the choice as either HPN or death. In implementing the decision, the principal mutable barrier to a timely discharge was communication difficulties across professional disciplines and organisations. Facilitators included developing a single point-of-contact between organisations, improving communication and implementing standardised processes. Oncologists and patients differ in their perceptions of how treatment decisions are made. Although patients are satisfied with the process, it might be beneficial for healthcare professionals to check patients’ understanding of treatment.

## 1. Introduction

Home parenteral nutrition (HPN) is the only method through which individuals with malignant bowel obstruction can receive adequate nutrition. There are indications that HPN might have benefits for patient well-being, however, robust evidence for improved quality of life or survival is lacking [1,2]. Nonetheless, the incidence of patients with advanced cancer—particularly ovarian cancer receiving HPN is increasing [3]. Currently, guidelines suggest that initiating HPN in advanced cancer should be based on expected survival, performance status and individual preferences [4]. Studies investigating survival and quality of life in those with advanced cancer on HPN have echoed this, emphasising that decision-making for starting the treatment is complex and should take into account a patient’s beliefs and personal preferences [5]. 

Currently, there is a paucity of research on how the decision to initiate HPN in patients with palliative needs is taken; albeit one study evaluated HPN in patients with advanced cancer [6]. Here, experienced nurses providing home care suggested HPN due to patients’ limited oral intake. Patients and their relatives were receptive to this recommendation, but reported that the decision for HPN was taken jointly between nurses, patients and relatives. However, these patients differ from those in bowel obstruction, as HPN was supplementary to their oral intake and not the sole source of nutrition. 

Once a patient has agreed to HPN, the subsequent process involves liaison between multiple healthcare professionals, patients and their families. Discharge arrangements need to be coordinated in a timely manner, as such patients have limited survival. Discharging patients with palliative needs can be challenging due to the complexities of arrangements required [7]. Hence, an understanding of the barriers and facilitators surrounding these complex discharges would potentially inform future practice, leading to more streamlined services. 

The aim of this study was to explore the decision-making process for HPN in patients with ovarian cancer in bowel obstruction and to investigate barriers and facilitators to implement the decision. 

## 2. Materials and Methods 

This was a qualitative study underpinned by phenomenology; a method that allows description and interpretation of complex processes. This study forms part of a programme of work examining parenteral nutrition in advanced ovarian cancer; patients’ views and clinical details have been reported elsewhere [8,9,10]. 

### 2.1. Participants 

Patients with ovarian cancer admitted to hospital with bowel obstruction between October 2016 and December 2017 considered for HPN, their relatives and healthcare professionals involved in their care were invited to participate. All participants gave written informed consent and ethical approval was granted by the East of England Cambridge Central Research Ethics Committee, Reference 16/EE/0330.

### 2.2. Context

**Figure 1:**
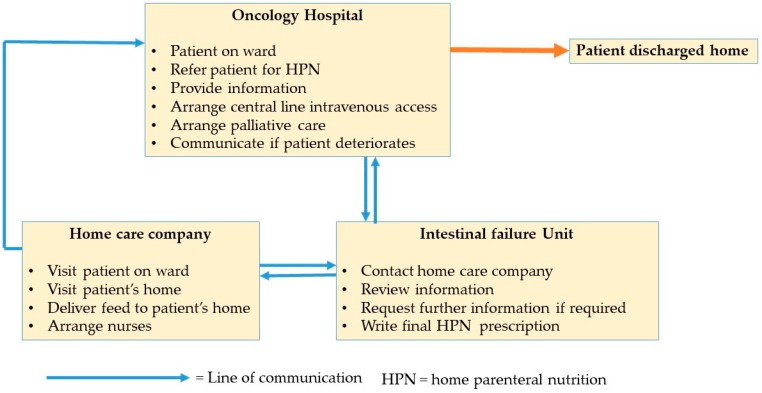
Pathway for patient discharge on home parenteral nutrition.

Patients were started on parenteral nutrition at a specialised oncology hospital. HPN discharge was overseen remotely by a specialist intestinal failure unit (IFU) situated in another hospital (Figure 1). All discharge arrangements for HPN followed a remote discharge pathway agreed between the two hospitals and developed over a number of years [11]. The IFU contracted a homecare company to supply equipment and feed to the patient at home. The homecare company also employed nurses to administer feed.

### 2.3. Data Collection

Longitudinal in-depth interviews were carried out. Participants were interviewed up to four times; patients and relatives at hospital or their home and healthcare professionals at their workplace. Different interview topic guides were used flexibly for each participant group (See supplementary material—A: Patient interview guide, B: Healthcare Professional interview guide and C: Relative interview guide), allowing the interviewer to follow up on issues raised by the interviewee. With participant permission, interviews were audio recorded, encrypted at source and transcribed verbatim.

Data collection continued until saturation of themes was reached. This was established through concurrent data analysis, which is an iterative process of reading and analysing transcripts as interviews proceed.

### 2.4. Data Analysis 

Interview transcripts were analysed thematically, guided by the techniques of Van Manen [12]. Transcripts were read repeatedly and reading progressed from considering the interview as a whole, to selecting phrases, to a detailed line-by-line coding. Themes were produced from the codes and similar themes were grouped together to form a hierarchy of themes and subthemes. Themes have been illustrated with quotations from the transcripts, giving some key points of reference; patients are identified by pseudonyms and age at first interview, healthcare professionals are identified by their job role and the relatives are identified by pseudonyms and relationship to the patient. The process was managed using the NVivo Version 11 (QSR International Pty Ltd., Doncaster, VIC, Australia). 

Rigour was introduced by two researchers (AMS and SB), developing the coding framework and separately double-coding a sample; any discrepancies were resolved by discussion. Overall coding framework was discussed with ES. The interviewer (AMS) kept field notes and used them as a space for reflexivity. The longitudinal nature of the interviews allowed for clarification of meaning, which facilitated some degree of member checking [13]. 

## 3. Results

During the study period, 26 patients with ovarian cancer and bowel obstruction were considered for HPN, all patients were invited to participate and 20 patients agreed to take part. Patients had a mean age of 67 years (SD 7.5 years). In addition, 13 relatives and 32 healthcare professionals from a variety of disciplines took part (Table 1).

### 3.1. Decision to Start Home Parenteral Nutrition

The decision for HPN was driven by oncologists who made a judgement informed by patient life expectancy and performance status regarding suitability. Oncologists reported that they discussed HPN with patients that were deemed suitable and the individual made their own decision to have artificial feeding or not: “*It’s about having that discussion with the patient about the various options available and trying to help the patient make their own individualised decision of what we feel is in their best interest. So we would say there is [HPN]. These are the pros and cons of [HPN]*” (Oncologist).

On being offered HPN, the patients presented an alternative view reporting the doctor had recommended it or had made the decision based on clinical necessity. One of the dietitians commented that some patients wanted to put the onus on the doctor to make the decision; “*I do think a lot of patients… think to some degree if it’s something that I need then I’ll do it, let the doctor make that decision*” (Dietitian). Both patients and oncologists highlighted that patients would not have been aware of HPN if they had not been offered it in the first place. 

Although patients did not feel they made the decision, no one reported they were coerced into HPN. This was possibly because these patients felt there was no decision to be made; “*there was no choice really, it was one of those take it or leave it, they didn’t say that, but it’s a take it or leave it, isn’t it*?” (Susan, 73). Another patient commented: “*Well, to me it was a no option thing. I don’t think they could have done anything else, bar starve me… if that’s what’s keeping me alive, it’s what I have to have isn’t it. So I don’t think [there was] a decision as such, if there was no other… if I can’t eat, it will be next best thing*” (Brenda, 70). One woman referred to it as “*Hobson’s choice*”, (Hilda, 62), which summed up the thinking style of these patients. For them, the choice for HPN was a stark one of having the feed and staying alive or dying.

Other members of the multidisciplinary team had a peripheral role. A complex care nurse viewed the dietitians and doctors as making the decision; “*it’s the dieticians and the medical team, to decide and see if they feel* [HPN is] *appropriate*”. Dietitians did not see themselves in this light. They would raise the question whether a particular patient should be considered for HPN with the doctors. However, they would not mention HPN to a patient without a consensus within the team regarding therapeutic and supportive options, as they did not want to cause problems or as two dietitians independently said, “*open a can of worms*”. (Dietitian 1; Dietitian 2). The palliative care nurses interviewed were supportive of HPN “*you are… giving precious time to people when there are things that they want to do and [HPN] allows them to do that*” (Palliative care nurse). However, they generally had no involvement in HPN decision making, apart from discussing decisions made by others in cases where patients were discharged without it.

Five patients were not discharged on HPN although they received artificial feeding in hospital. For most, this was due to clinical deterioration and a judgement being made that HPN would not benefit them; “*I saw* [consultant] *on Monday and he said it may be stopped if the benefits are outweighed by the disadvantages, so I’m just leaving it to him*” (Margaret, 69). One women declined HPN as she felt she had been in hospital long enough. Her family seemed to be instrumental in the decision as her daughter-in-law was not keen on the patient having it. 

### 3.2. Barriers to Timely Discharge

Healthcare professionals discussed a number of barriers to a timely and safe discharge. One immutable barrier was the complex needs of patients due to multiple medical problems that could become unstable within a short timeframe, delaying discharge; “*unfortunately our patients aren’t always straightforward*” (Nurse).

Poor communication between healthcare professionals was a potential barrier, given the number of people involved. One patient commented on the number of professionals she had seen “*there’s that many people been, that many doctors, that many therapists*” (Brenda, 70). Communication was particularly important, as healthcare professionals came from different disciplines with different priorities and calls on their time. However, their actions were interlinked, each professional group relied on others to act to enable a timely discharge; “*it’s the start bit that takes the time… at least fill the form in… I know [doctors are] busy, but… the referral process doesn’t start until IFU… receive [the form]*” (Nurse).

Complexities also arose due to a liaison being necessary between the organisations based at three different sites (IFU, oncology hospital, and the homecare company). Distance meant that communication was via email or telephone and initially lacked continuity, with a number of staff involved at each site. As one home company nurse said “*I think if we had a link within the [oncology hospital], if we had one person… to go to*”. Moreover, as the homecare company were contracted by the IFU, all communication flowed through there. However, the IFU manager reported this was not always the most efficient way of dealing with small issues; “*sometimes I’ve said the homecare company and [oncology hospital] should communicate together… I’ve said just go direct and then just let me know what’s happening*” (IFU manager).

### 3.3. Facilitators

Facilitating the best service for patients was the reason for the development of remote HPN discharge; “*if we were to send them over to* [IFU], *it’s… another hospital for the family to go to*” (Dietitian). Since the service started, all involved worked to reduce the discharge time; “*I think it’s really good the way the process has changed… we used to have patients sat in for six to eight weeks waiting for it… But two weeks is quite amazing for me*” (Palliative care nurse).

Healthcare professionals discussed measures put in place over the years, which might have reduced discharge time, such as improved communication between staff. This occurred by increasing opportunities for face-to-face meetings. At the oncology hospital, dietitians joined weekly ward round meetings with the oncologists. This enabled them to have updated information on patients and they could provide feedback on any nutritional problems. An additional benefit was improved communication and strengthened relationships between dietitians and doctors: “*Our communication with the [medical] team has improved over the years… we try to go every week to the… ward round… it’s just more open dialogue with them*” (dietitian). 

Improved communication between the two hospital sites was also a side benefit of developing an increasingly standardised process for HPN discharge. A pathway was established at service inception, but was refined over the years. During this study, the two hospitals were working towards a more formalised arrangement with a service level agreement to fit in with NHS tariffs. This required more regular meetings between staff from the two hospitals, facilitating relationships and communication. Moreover, it gave a forum to discuss and resolve difficulties. 

An enabling factor that had a big impact on timely discharges was the appointment of a nutrition nurse at the oncology hospital during the course of the study. She liaised with a manager at the IFU: “*So the idea was that I would streamline it all, I would be the co-ordinator at this end, and [IFU manager] would be the co-ordinator at that end, and everything would go through me and her, unless there was a specific query that only the dietitian or a doctor could answer… it has, on the whole, improved. We’ve got it down on a straightforward patient to… a two to three week maximum turnaround time*” (Nurse). Although devolving responsibility to key personnel at the two sites was an enabler to timely discharge, it became a barrier when one retired or was absent on vacation time. 

### 3.4. Patient Perceptions of the Process

Shortening time to discharge was a key priority for staff as this was what they perceived that the patients wanted; a correct assumption in most cases. Patients knew that they had a short time left and wanted to return home as soon as possible. They found it difficult to wait two weeks for HPN to be organised. “*I’ve been nagging them about going home… I’ve been in here long enough… it could be another two weeks, which I’m not happy with but there’s nothing I can do*” (Ophelia, 77). However, generally patients understood that arrangements needed to be made before they could go home: “*I don’t know what the process is but you can’t just go home. It’s got to be in place… and somebody would have to come… to administer it… it does take at least ten days to get it organised* “(Catherine, 60).

For some patients, the time from the decision for HPN to discharge was too quick. This was particularly so for the frailer patients who lived alone or had been in hospital for a long time. As one said, “*I felt it was just moving a bit too quick at the end… I just felt a lot was being pushed on* [Daughters*] in those last few days*” (Brenda, 70). Brenda’s daughter thought that her mother was concerned about her and her sister, but also she was wary of coming home: “*I think she’s scared of coming home. As much as she wants to, she’s been in there for three months… and then she’s going home just to herself. And me. She’ll go from having 24-h care to not… But she’s making it that she’s concerned about me and [sister] not having everything ready* “(Adele, daughter).

One patient whose discharge was delayed due to medical reasons was pleased to have more time in hospital and was reassured when no issues came up on the ward: “*I’m possibly not as worried as I was in the beginning about it… with the machine… there’s been no problems once it’s up… Yeah, I’m getting less apprehensive about that*” (Mabel, 72). “*So maybe there is a benefit in being in the hospital a little bit*” (Interviewer). “*Oh, a real benefit*” (Mabel, 72).

## 4. Discussion

Increasingly patients with advanced ovarian cancer are having HPN, so it is essential to understand if patients are satisfied with the decision making process and the barriers and facilitators to implement the decision [3]. In this study, we found variance between oncologists and patients regarding ownership of the decision for HPN. However, patients who were discharged on HPN were content with the decision for artificial feeding. Once the decision was made, a complex process ensued involving multiple professionals in different locations to operationalise the decision to provide HPN.

There has been a move to promote patient involvement in decision making, as a way of upholding patient-centred care [14]. In general, doctors have been supportive of shared decision-making [15]. The oncologists in this study viewed themselves as providing a space for shared decision-making and reported that their patients were actively involved in decision-making. The patients, however, thought the oncologist had made the treatment decision, although they were happy to accept their suggestion, which resonated with other studies; for example, Ciambrone interviewing women about their treatment choices in breast cancer found the majority left treatment decisions to their doctor [16]. In our study, although there was a difference in perception between doctors and patients, it could be questioned whether this was problematic, as all patients who went home on PN were content to have the treatment. Moreover, it is apparent that patients were not coerced into treatments, as there was an instance where HPN was declined. Furthermore, not all patients want the responsibility of actively being involved in treatment decision making [17]. However, given this disparity of perception it might be worth it for clinicians to check patient understanding of the decision and the implications of its implementation, as HPN involves nurse visits and equipment in the home. 

Another reason for the divergence in perception might be due to patients reporting that there was no choice to be made, as reported elsewhere [18,19]. Ziebland et al. and Charles et al. found patients commenting that there was no decision to be made if there was only one treatment option [18,19]. The patients in the current study viewed the alternatives as HPN or death. It has been argued that patients seeing themselves in this situation do not view the choices equally [20]. Patients make the choice to live and then by necessity accept whatever they perceive will facilitate this, in this case HPN. 

The multidisciplinary team also viewed the oncologist as making the decision for HPN. Multidisciplinary working is now common place in the health service and there has been some flattening of the traditional medical hierarchy where the oncologist is in a position of dominance and other healthcare professionals are subordinates [21,22]. However, the medical hierarchy still exists to a certain extent; allied health professionals, like the dietitians in this study, can be circumspect about discussing treatment options with patients, for fear of “opening a can of worms” [23]. 

Once the decision has been made, it needs to be implemented. Good communication and a standardised process are two elements identified as improving patient discharge [24]. These two elements seemed to have facilitated the reduction in time to discharging patients on HPN. 

Discharging a patient on HPN is a complex process requiring multiple healthcare professionals in different professional disciplines. Good communication across those disciplines is difficult in the modern healthcare environment, with the multiple time demands on professionals [25]. It has been noted that a patient might be dealing with many different healthcare professionals in a day [26]. This means liaison is imperative for good patient care and the more opportunities, either formal or informal, that staff have to interact, the better the collaboration and communication is between them [26]. Multidisciplinary team meetings have been identified as the key mechanism for communication across professions, and dietitians particularly discussed enhanced communication within the team after they started attending the weekly ward round meeting [25]. Appointing a nutrition nurse as a single point-of-contact at the oncology hospital also improved communication and reduced time to discharge. The nurse was acting as a discharge co-ordinator for HPN, a role recommended by National Institute for Health and Care Excellence for patients with complex needs [27]. 

Discharge protocols have also been shown to reduce discharge time [28]. The team had a standard pathway and working to refine it had the dual benefit of greater standardisation and improved communication, as this process necessitated regular meetings between staff from the two hospitals.

## 5. Conclusions

In conclusion, oncologists and patients have different perceptions of how treatment decisions are made. However, this does not mean that patients are dissatisfied or feel coerced into treatments, although it might be beneficial for oncologists to check that patients understand what HPN involves. Once a treatment decision has been made, good communication and standard care pathways facilitate smooth implementation.

## Figures and Tables

**Table 1:** Numbers of relatives and healthcare professionals participating.

**Relatives**	**Numbers**
Husband	8
Daughter	4
Son	1
Total	13
**Healthcare Professionals**	**Numbers**
Oncologist	4
Gastroenterologist	1
Homecare Nurse	5
Dietitians	9
Dietetic Manager	1
Doctors (junior and senior)	2
Supportive and Palliative care nurses	2
Complex discharge nurse	1
Advanced nurse practitioner	2
Nutrition support nurse	1
Homecare nursing managers	3
Intestinal failure unit manager	1
Total	32

## Supplementary Materials

The following are available online at https://www.mdpi.com/2072-6643/12/1/166/s1, A: Patient interview guide, B: Healthcare professional interview guide, C: Relative interview guide.
