# Supplementary material for: Discharging Women with Advanced Ovarian Cancer on Home Parenteral Nutrition: Making and Implementing the Decision
Source: Nutrients. 2020 Jan 7;12(1):166. doi: 10.3390/nu12010166 (PMC7019843; doi:10.3390/nu12010166)
Supplement: Supplementary file 1 [file nutrients-12-00166-s001.zip › Appendix A Patient interview topic guide.docx]

**Appendix A: Interview Topic Guide (Patient)**

1. Tell me the story of how you came to have feeding into a vein

Probe: Was it your decision or did the doctor decide for you?

What did you think about when making your decision?

What were the pros?

What were the cons?

Which health care professionals discussed feeding into a vein with you?

- What did they say?

Did you discuss your decision with family or friends?

- If so, what did they say?

1. What are your experiences of feeding into a vein so far?

Probe: How do you feel about it?

What are the benefits of the treatment?

What are the disadvantages of the treatment?

What do you hope it will achieve?

Is it what you expected before you started it?

- can you tell me more about that?

Have your symptoms improved or not? Can you tell me more?

1. Tell me about your food and drink intake recently

Probe: Has that increased or decreased since starting feeding into a vein

1. What are your thoughts about having feeding into a vein at home?

Probe: benefits/disadvantages of treatment at home

1. Do you have anything else you would like to tell me?

Interview 2

1. Tell me about the transition on feeding from the hospital to home

Probe: What was the process like of setting up for home?

- Time to arrange

- Communication different staff members between themselves

- Communication between staff and you

- Set up visit from homecare nurse

What are your thoughts on having the equipment for feeding at home?

What are the benefits/advantages of the treatment?

What problems or disadvantages have you encountered?

Have your symptoms improved or not? Can you tell me more?

How do you feel about feeding into a vein now?

Is having feeding into a vein at home as you thought it would be or not? Can you tell me more?

What factors did you think about in making the decision to have feeding into a vein at home?

Is there anything you wished you had been told about in the hospital?

1. Tell me about any involvement of health care staff with your feeding into a vein?

Probe: Do staff visit you at home?

– if so, which staff come?

– What do they do?

Do you attend out patient’s clinic?

1. Tell me about your experience of the feeding company?

Probe: Any problems or not?

- Deliveries
- Staff
- Contact numbers

1. Do you have anything else you would like to tell me?

Interview 3 (& 4)

1. Tell me about your experience of feeding into a vein since we last met

Probe: any impact of having feeding into a vein on various aspects of your life or not?

- Physical health – symptoms?
- Relationships and social interaction
- Emotions

Any problems with the treatment or not?

- Any line infections
- Any hospital admissions

Any problems with the feeding company or not?

- Deliveries
- Staff

1. Overall what are your feelings about the treatment?

Probe: What are the benefits of the treatment?

What are the disadvantages of the treatment?

1. Has the decision to have feeding into a vein been a good one or not?

Probe: If yes why, if no why

Having experienced feeding into a vein – would you make the same decision to have the treatment?

- Can you tell me more about that?

Have you thought about stopping feeding into a vein?

- Can you tell me more about that?

1. Do you have anything else you would like to tell me?
