# Supplementary material for: Discharging Women with Advanced Ovarian Cancer on Home Parenteral Nutrition: Making and Implementing the Decision
Source: Nutrients. 2020 Jan 7;12(1):166. doi: 10.3390/nu12010166 (PMC7019843; doi:10.3390/nu12010166)
Supplement: Supplementary file 1 [file nutrients-12-00166-s001.zip › Appendix B Healthcare professional interview topic guide.docx]

**Appendix B: Healthcare professional interview topic guide**

1. Could you briefly tell me about your role and your involvement with this patient?
2. What factors did you consider in recommending parenteral nutrition on the ward to this patient?

Probe: What are the benefits of the treatment for them?

What are the disadvantages?

1. What did the patient say about your recommendations?

Probe: What were their main concerns or worries?

Did you involve their relatives in the decision as well – why or why not?

1. Have there been any adverse events related to parenteral nutrition so far?

Probe: line infections / fluid balance / electrolytes?

1. Are you going to recommend home parenteral nutrition?

Probe: If so why/ if not why?

1. If parenteral nutrition has been recommended already – what did the patient say?

Probe: Were relatives involved – why or why not?

1. Is there anything else you would like to add?
