# Supplementary material for: Discharging Women with Advanced Ovarian Cancer on Home Parenteral Nutrition: Making and Implementing the Decision
Source: Nutrients. 2020 Jan 7;12(1):166. doi: 10.3390/nu12010166 (PMC7019843; doi:10.3390/nu12010166)
Supplement: Supplementary file 1 [file nutrients-12-00166-s001.zip › Appendix C Relative interview topic guide.docx]

**Appendix C: Relative interview topic guide**

1. Tell me a little about yourself
2. Tell me the story of how your relative/friend came to have feeding into a vein

Probe: Did they discuss the decision with you?

– if so what did they say?

Did any health care professionals involve you in the decision?

- if so what did they say?

What do you think about the decision?

1. What has been your experience of having your relative of feeding into a vein up until now?

Probe: How do you feel about it?

What do you hope feeding into a vein will achieve for your relative?

1. Tell me about how they have been eating and drinking recently

Probe: Has that increased or decreased since starting feeding into a vein

1. What are your thoughts about your relative having feeding into a vein at home?

Probe: Do you think there will be any impact on you?

– Can you tell me more about that?

Do you have any concerns about your relative going home on feeding into a vein or not?

- if so, can you tell me more about that?

For you, what do you think will be the advantages?

For you, what do you think will be the disadvantage?

1. Do you have anything else you would like to tell me?

- Interview 2

1. Tell me about the transition on feeding from the hospital to home

Probe: Can you tell me how you have experienced the process

How has your relative/friend having feeding into a vein impacted on you?

- Can you tell me more about that?

Is your relative/ friend having feeding into a vein at home as you thought it would be?

Any changes in your relationship with the patient since they started feeding into a vein or not?

What have been the benefits/advantages of the treatment?

What problems or disadvantages have you encountered?

What was the process like of setting up for home?

- Time to arrange

- Communication different staff members between themselves

- Communication between staff and you

- Set up visit from homecare nurse

What are your thoughts on having the equipment for feeding at home?

1. If carer trained in giving feed – what was your experience of the training?

Probe: What was good about the training?

What could have been improved with the training?

Did the training equip you to give your relative feeding at home or not?

1. Tell me about the involvement of health care staff with your relative’s feeding into a vein?

Probe: Do staff visit your relative/friend at home?

– if so which staff come and what do they do

Is the level of help you receive the right amount or not?

– can you tell me more?

Does your relative/friend attend out patient’s clinic?

1. Have there been any problems with the feeding company or not?

Probe: How has this impacted on you?

1. Do you have anything else you would like to tell me?

Interview 3 (& 4)

1. Tell me about your experience of your relative/friend having feeding into a vein since we last met

Probe: Any impact on various aspects of your life or not?

- Physical health
- Relationships and social interaction
- Emotions

Any problems that your relative/friend had with the treatment or not?

- Any line infections
- Any hospital admissions

Any problems with the feeding company?

- Deliveries
- Staff
- Contact numbers
- How has this impacted on you?

1. Overall what are your feelings about the treatment?

Probe: What have been the benefits of the treatment?

What have been the disadvantages of the treatment?

1. Has the decision for your relative/friend to have feeding into a vein been a good one or not?

Probe: If yes why, if no why

Have you thought that your relative should stop feeding into a vein?

- Could you tell me more about that?

1. Do you have anything else you would like to tell me?
